# Critical Role of Organic Spacers for Bright 2D Layered Perovskites Light‐Emitting Diodes

**DOI:** 10.1002/advs.201903202

**Published:** 2020-02-19

**Authors:** Hsinhan Tsai, Cunming Liu, Eli Kinigstein, Mingxing Li, Sergei Tretiak, Mircea Cotlet, Xuedan Ma, Xiaoyi Zhang, Wanyi Nie

**Affiliations:** ^1^ Material Physics and Application Division Los Alamos National Laboratory Los Alamos NM 87545 USA; ^2^ X‐ray Science Division Argonne National Laboratory Lemont IL 60439 USA; ^3^ Center for Functional Nanomaterials Brookhaven National Laboratory Upton NY 11973 USA; ^4^ Theory Division Los Alamos National Laboratory Los Alamos NM 87545 USA; ^5^ Center for Nanoscale Materials Argonne National Laboratory Lemont IL 60439 USA

**Keywords:** charge localization, light‐emitting diodes, Ruddlesden–Popper layered perovskites, X‐ray absorption spectroscopy

## Abstract

Light‐emitting diodes (LEDs) made with quasi‐2D/3D and layered perovskites have undergone an unprecedented surge as their external quantum efficiency (EQE) is rapidly approaching other lighting technologies. Manipulating the charge recombination pathway in semiconductors is highly desirable for improving the device performance. This study reports high‐performance layered perovskites LEDs with benzyl ring as spacer where radiative recombination lifetime is longer, compared with much shorter alkyl chain spacer yields. Based on detailed optical and X‐ray absorption spectroscopy measurements, direct signature of charges localization is observed near the band edge in exchange with the shallow traps in benzyl organics containing layered perovskites. As a result, it boosts the photoluminescence intensity by 7.4 times compared to that made with the alkyl organics. As a demonstration, a bright LED made with the benzyl organics with current efficiency of 23.46 ± 1.52 cd A^−1^ is shown when the device emits at a high brightness of 6.6 ± 0.93 × 104 cd m^−2^. The average EQE is 9.2% ± 1.43%, two orders of magnitude higher than the device made with alkyl organics. The study suggests that the choices of organic spacers provide a path toward the manipulation of charge recombination, essential for efficient optoelectronic device fabrications.

## Graphical abstract

The charge recombination pathways in layered perovskites are closely related to the choice of the organic spacers. Using phenylethylamine as organic spacer greatly extends the charge recombination lifetime by localizing the hole near the band edge. Efficient Ruddlesden–Popper layered perovskites light emitting‐diodes are thus demonstrated.

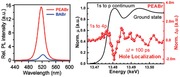

Ruddlesden–Popper phase layered perovskites (RPLPs) are quantum‐wells consisting of MX_6_ perovskites octahedron cages sandwiched between large organic spacers.^1^, ^2^ Because of the low‐dimensional layered structures, RPLPs possess many unique optoelectronic properties that are different from their 3D counterparts. For example, the optical band gaps widen with decrease of the number of octahedron‐layers between the organic spacers due to quantum or dielectric confinement effect.^3^, ^4^ More recently, surface states are discovered that are attributed to the local structural distortion of the layered perovskites.^5^ Motivated by the high emission quantum efficiency and large tunability in the optical properties, many efforts were devoted to the fabrication of light emitting diodes (LEDs) using quasi 2D/3D perovskites^6^, ^7^, ^8^ and low‐dimensional perovskites.^9^, ^10^, ^11^, ^12^, ^13^, ^14^ The typical quasi 2D/3D and low‐dimensional perovskite based LEDs output high brightness of 10^3^–10^5^ cd m^−2^ along with external quantum efficiency of 10–20%.^9^, ^12^, ^15^, ^16^ The emission mechanisms underpinning such high performances, have been attributed to various physical origins. For example, it is proposed that the high binding energy of excitons in the low‐dimensional perovskites is playing a significant role that promotes the radiative recombination leading to a high emission quantum yield.^17^ Other studies have attributed the efficient emission to the energy landscapes formed by different quantum wells thickness (or *n*‐number) across the film that cascade the charge carriers to the lowest energy emissive sites for recombination.^14^ In addition, the electron‐phonon couplings are found to be important in promoting the recombination pathways^18^, ^19^ as evidenced by Raman spectroscopy characterizations that suggest the design guidance of layered perovskite for emission applications.^18^

Here, we show that the charge recombination pathways in RPLPs are closely related to the material steric hindrance. By selecting benzyl organic spacers in the layered perovskite structures, we demonstrate ≈70 times brighter light emitting diodes (LEDs) than those made with linear organics. These superior LED's superior output luminance efficiency approaching 25 cd A^−1^ and champion external quantum efficiency of over 9%. To understand the emission mechanisms, we first investigated the photo‐physical properties of the thin films using photoluminescence (PL) spectroscopy. We found the carrier lifetime to be 5 times longer for the 2D perovskite thin films with benzyl ring than the films comprised with alkyl chains (**Figure** **1**a), which increases the ration of radiative recombination and thus yields 7.4 times higher PL quantum yield in the former. We further probed the photo‐induced electronic band structure evolution using time‐resolved X‐ray absorption spectroscopy. In these experiments, we observed a direct signature of hole localization around Br p‐orbital in the RPLPs with benzyl organics, whereas the signal is absent in the film made with alkyl organics because of the rapid carrier decay in the later. From those detailed spectroscopic characterizations, we conclude that in the RPLPs with benzyl organics, charges are getting localized near the band edge with prolonged carrier lifetime, which altogether enhance the radiative recombination probability. In contrast, in the thin film with alkyl chains, charges live much shorter and are likely to undergo efficient non‐radiative decay rapidly that reduces the emission efficiency. As a consequence, the LEDs made with RPLPs thin films consisting of benzyl chain shows high probability of emission leading to a high luminance over 7.51 × 10^4^ cd m^−2^. A detailed device characteristics analysis suggests that the carriers injected in the benzyl chain perovskite films recombine and emit light efficiently at a lower threshold voltage, and output a highest current efficiency of 25 cd A^−1^. This is a direct consequence of the observed long PL lifetime of localized charges near the band edge. Our study suggests materials design principles for emissive RPLPs by tuning organic spacer moiety controlling charge localizations and carrier recombination toward high efficiency light emitting diodes.

**Figure 1:**
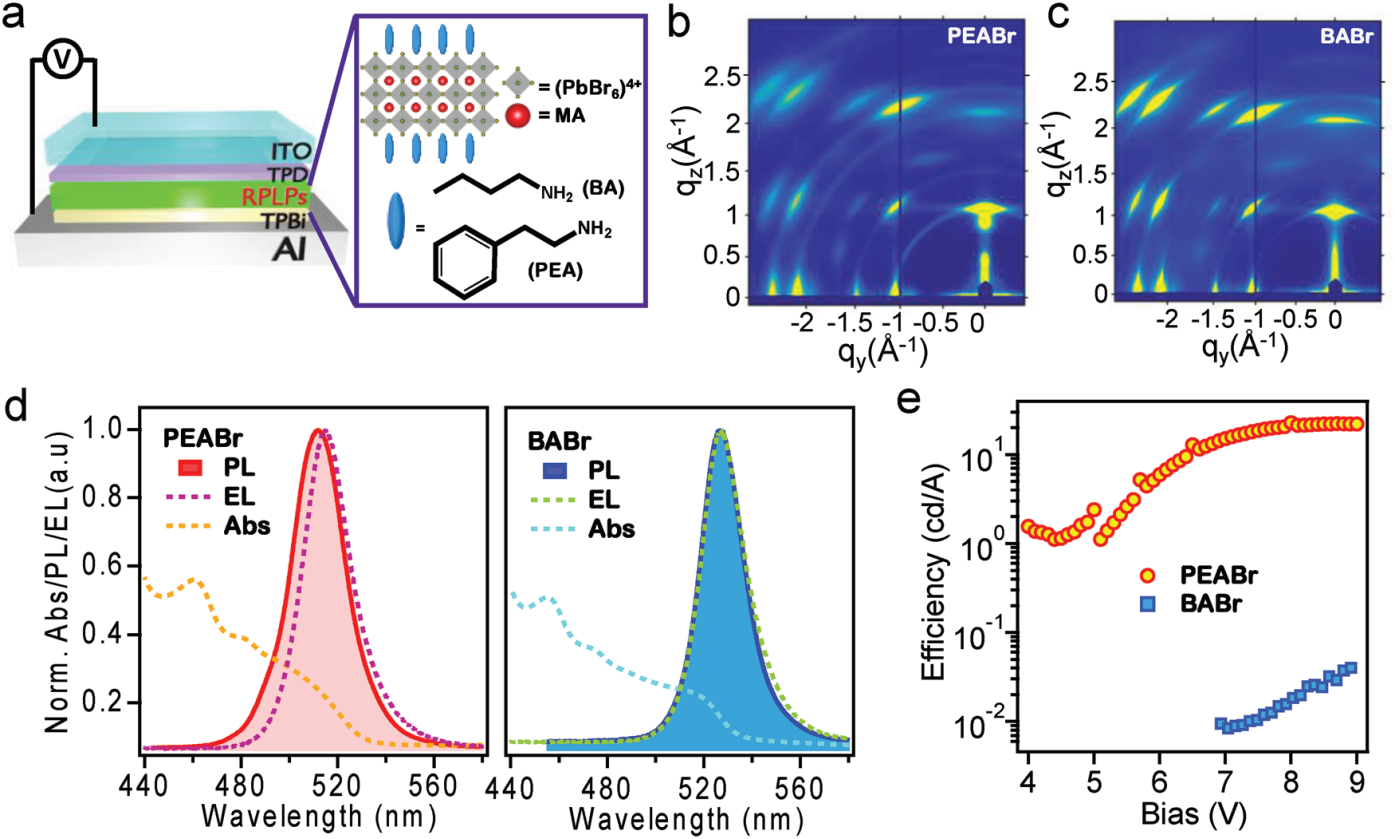
Spectroscopic and optoelectronic properties of 2D perovskites. a) Schematic illustration of the LEDs device architecture and layered perovskite material structures with butylamine (BA) and phenylethylamine (PEA) as large organic spacers. Thin‐films GIWAXS maps for b) PEABr and c) BABr. d) The absorption, photoluminescence, and electroluminescence obtained from PEABr (left) and BABr (right) layered perovskite thin films and devices. e) The LEDs' current efficiencies as a function of the bias for PEABr and BABr devices.

We use layered perovskite materials following the structure of R_2_MA*_n_*_−1_Pb_n_Br_3_ *_n_*_+1_ to make the light LEDs. Figure 1a shows the LED's device architecture used in this study, the 2D perovskite material structure, and the chemical structure of the two compounds chosen for this work. Here, the organic spacer R is chosen to be either butylamine (BA) or phenylethylamine (PEA). We keep the same quantum‐well thickness (*n* = 3) in 2D RPLPs and utilize the same thin film deposition conditions. We first examine the intrinsic materials structure by power X‐ray diffraction patterns (PXRD) of as‐synthesized single crystals for BA_2_MA_2_Pb_3_Br_10_ (BABr) and PEA_2_MA_2_Pb_3_Br_10_ (PEABr) (see Figure S1 and the detail discussion in Supporting Information) The PXRD data confirm and show the well‐resolved, equally spacing (0*k*0) peaks for three octahedron layers (*n* = 3) in low angle regime for both PEA‐perovskite and BA‐perovskite which suggest the phase purity of these materials.^1^, ^20^ After validating the PEA‐perovskite and BA‐perovskite structures, we deposited these two compounds into thin films by employing the same preparation approach (see Experimental Section) for thin film crystal structure characterizations. Figure 1b,c are the synchrotron grazing incidence wide‐angle X‐ray scattering (GIWAXS) maps collected from those two thin‐films (see detail in Supporting Information). The GIWAXS maps line‐cut and FWHM data (see Figure S2, Supporting Information) show similar feature in both PEABr and BABr thin films with discrete Brag diffraction patterns indicating both have high degree of crystallinity and comparable intensity. We notice that the peak position and broadening of both films are similar, suggesting the crystallinity and crystal orientation in PEABr and BABr thin films are comparable.^4^, ^13^, ^21^ We further examined the surface and cross‐section of the two films by scanning electron microscopy (see Figure S3, Supporting Information) and both of the samples show similar surface morphology and crystalline structures in the film.

Figure 1d shows the thin film absorption, photoluminescence (PL) and electroluminescence (EL) spectra obtained from PEABr and BABr films and devices. The PL spectra overlap with the absorption band edges reasonably well in both films, and no significant Stokes shift is observed. Moreover, the PL spectra of PEABr and BABr thin films exhibit photoemission peaks at 511.4 and 526.4 nm with FHWM of 28.0 and 22.3 nm, respectively. We also notice that the EL peak is slightly red shifted (515.1 nm) from the PL peak by 4 nm in the PEABr, whereas the BABr's EL peak (526.9 nm) overlaps well with its PL spectrum. The red shift of the EL peak versus PL peak has been previously attributed to an electric‐field‐induced Stark effect.^6^, ^22^ When comparing the absorption and PL between the two films, we notice the BABr films show 15 nm red shift than that of PEABr. It is demonstrated in the literature that the mixed phase of various *n*‐numbered 2D perovskites can affect the emission properties. In our case, the stronger shoulder in BABr thin film from the absorption spectrum suggests the presence of a mixed phase from higher *n*‐numbered (or 3d) perovskite phase.^14^

These PEABr and BABr thin films with comparable quality were further assembled into LEDs devices following a typical architecture illustrated in Figure 1a. The device characteristics are plotted in Figure 1e, where the current efficiency (in unit of cd A^−1^) is employed to assess the device performance. Figure 1e shows a typical current efficiency as a function of the applied bias curve for LEDs devices based on PEABr and BABr materials. The PEABr device reaches a peak current efficiency of 25 cd A^−1^ while BABr shows only 0.097 cd A^−1^. These results are reproducible for 4 batches of devices and we consistently observed such difference for those two devices. Moreover, the PEABr LED device turns on at lower voltage bias and rapidly reaches to the maximum efficiency. To understand such a drastic difference between the two types of LEDs, we probed their intrinsic emission properties of related thin films (**Figure** **2**a) by performing PL spectroscopy and microscopy (Figure 2).

**Figure 2:**
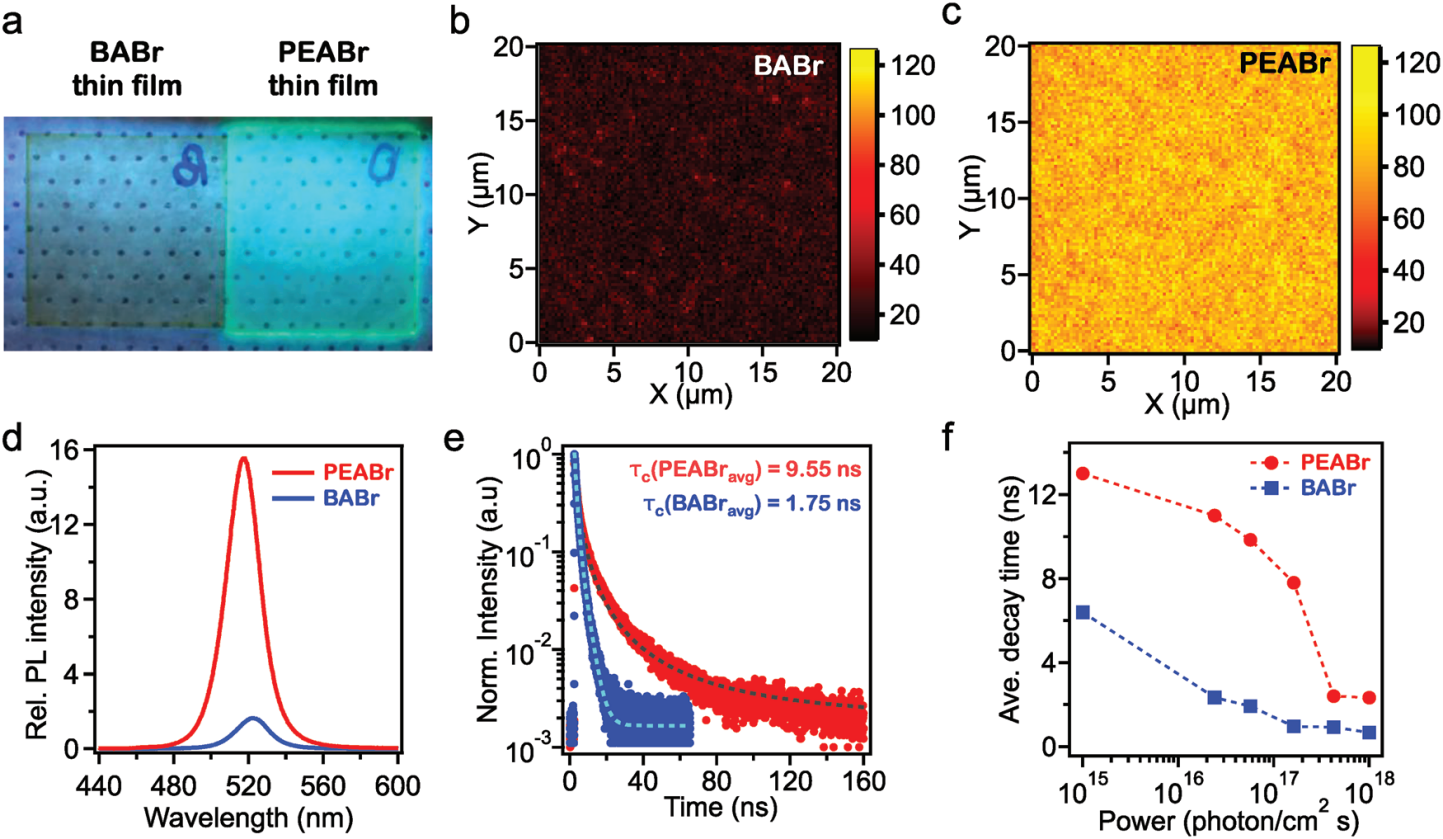
Optical spectroscopy and microscopy of 2D perovskites thin films. a) Photograph of PEABr and BABr thin films under UV (365 nm) exposure. b,c) High‐resolution PL maps for BABr and PEABr thin film, respectively. d) PL spectra for PEABr and BABr thin films recorded in identical conditions including identical laser power. e) Time‐resolved PL (TRPL) decay curves for PEABr and BABr thin films. Reported PL lifetimes are average values (see text fir details). f) Average PL decay times versus incident laser power.

Figure 2a shows the photograph of BABr (left) and PEABr (right) thin films under UV lamp (365 nm) irradiation. When comparing them visually side by side, the PEABr film glows while BABr film is much dimer under the same UV excitation. This is not due to the thin film morphologies (Figure S3, Supporting Information) which might create non‐uniform photoemission. We further excited these two films with a laser (CW 405 nm) for PL measurements to quantify the emission efficiencies Figure 2b,c shows the spatially resolved PL maps for BABr and PEABr, both recorded using identical scanning laser power (0.5 mW cm^−2^). The PL intensity for PEABr is strong and uniformly distributed across the scanned area (20 µm × 20 µm), whereas that for BABr PL intensity is much weaker and features local non‐homogeneous spots (Figure S4, Supporting Information). The relative PL quantum yields (PLQY) are taken by the PL intensity normalized by the absorption cross‐section of the films (see detail in Supporting Information). As a result, the PLQY of PEABr is 7.4 times higher compared that of BABr (Figure 2d). Thus, strong and uniform PL intensity in the PEABr thin film is consistent with the high electroluminescence efficiency of this system, indicating the high LED performance made with PEABr can be mainly attributed to the intrinsic photo‐physical properties from the materials, particularly efficient radiative recombination of charge carriers.

To gain insight into the carrier recombination processes, the time‐resolved photoluminescence (TRPL) decays of these thin films were measured and compared. Figure 2e,f displays the TRPL decays and extracted average carrier lifetimes as a function of laser excitation power (see Supporting Information for details on calculation of lifetimes). Notably, the average PL decays for PEABr is 2–7 times slower than that in BABr at the same excitation powers (the laser power corresponds to photon flux of 10^16^–10^17^ photon cm^−2^ s^−1^) as shown in Figure 2e. This suggests that the photo excited carriers in PEABr tend to live much longer before recombining compared to those in BABr sample. When fitting the TRPL curve for PEABr sample, several components were observed in the PL decay which is an indication that multiple recombination processes are involved during emission, the slower component is usually shallow trap assisted recombination.^23^, ^24^ The PL lifetimes as a function of excitation power are plotted in Figure 2f. At low excitation power, we observe longer lifetime in PEABr which rapidly drops when the power increases. The high PLQY and TRPL results in PEABr films suggest that, the shallow trap‐assisted radiative recombination dominates the light emission mechanism, carriers exchange between traps and band edge at room temperature resulting in longer radiative recombination life times (Figure 2f). With the increase in laser excitation power increases, less traps are available and electron‐hole recombination rapidly through radiative pathway, leading to shorter PL lifetimes.^25^, ^26^ Carrier recombination lifetime gets comparable for both samples under high excitation power due to the enhanced electron‐hole radiative recombination caused by the high population of excited carriers (Figure S5, Supporting Information). Interestingly, such power dependence is expected in systems with high free carrier density that increases the recombination rate.^24^ A previous study has shown that the recombination in 3D CH_3_NH_3_PbI_3_ perovskites is dominated by free electrons in the conduction band and free holes in the valence band.^26^ This observation along with the rather negligible Stark effect in our case (Figure 1d) illustrates that the recombination occurs at the band edge through free carriers.

Combining the results of PL spectroscopic and micorscopic experiments, including the power dependence shown in Figure 2, we conclude that the emission mechanism for PEABr is dominated by shallow trap assisted free carrier recombination when this material is excited at a range of 10^15^ to 10^18^ photons cm^−2^ s^−1^. The long carrier lifetimes in PEABr are due to the exchange between shallow trap and the band edge. On the other hand, BABr thin film has lower PLQY and shorter carrier lifetime compared to PEABr, indicating the carriers are mostly recombined through non‐radiative decay mechanisms. Note that the photon flux used for PL study, produces carried density roughly comparable to the number of electrons injected to the LEDs, which is thus relevant to the LEDs operation. The greatly extended carrier lifetime is therefore responsible for improved light emission.

To connect the photo‐induced charge carrier dynamics to the electronic fine structures, we have applied time‐resolved X‐ray absorption spectroscopy (TR‐XAS) to both materials.^27^ The basic principle of the TR‐XAS measurement is illustrated in **Figure** **3**a, where a laser pulse is used to initiate electron excitations from valence to conduction bands, creating charge carries (electron and hole pairs), followed by an X‐ray probe pulse at certain time delay to measure the electronic structure dynamics after photoexcitation (see detail discussion in Supporting Information). Figure 3b,c presents the ground state XAS spectra of PEABr and BABr (black curves) measured at Br K‐edge, where colored symbol lines are their corresponding difference XAS spectra before and after laser excitation (Abs_laser_on_(t)‐Abs_laser_off_). The Br K edge absorption measures the transition from Br 1s orbital to upper uncopied orbitals and continuum with p‐characteristics. The intense X‐ray absorption edge peaked around 13.48 keV corresponds to Br's 1s to p continuum transition. The ground state spectra of two materials are almost identical, suggesting similar electronic and local geometric structures near the bromide atom (i.e., local bond distortion). After photo‐excitation at 100 ps, the XAS spectrum of PEABr shows noticeable changes, while that of the BABr remains about the same. clear features in the difference curves (colored symbols in Figure 3b,c)

**Figure 3:**
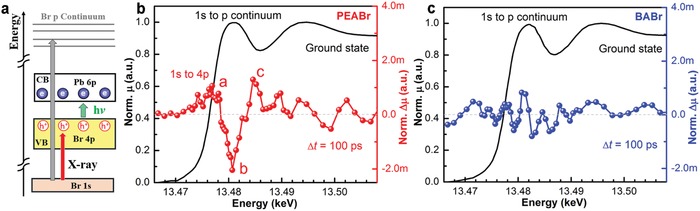
Light induced X‐ray absorption spectroscopy. a) Schematic illustration of bromide (Br) K near edge transitions. The ground state X‐ray absorption spectra as a function of energy and the change in X‐ray absorption after 100 ps laser excitation for b) PEABr and c) BABr thin films.

The difference spectrum of PEABr shows three clear features (labeled as a, b, and c in figure 3a): a positive peak around 13.4774 eV (a), a negative peak around the GS edge absorption peak (b), and then a positive absorption feature after the edge (c). Based on previous DFT calculation of bromide lead perovskite, the valence band has mainly Br 4p character.^28^ The excited Br 4p holes in the VB can be delocalized or localized through self‐trapping. The delocalized holes would not change XAS absorption transition energy too much while the localization of holes at Br center would shift the absorption edge to higher energy and also give rise to an extra transition from 1s to 4p holes in the VB. The positive feature at 13.477 eV in the different XAS spectra of PEABr comes from the transition from Br 1s to photon‐created 4p holes. Such transition only exists in the ES because the Br 4p orbitals in the GS are fully occupied. The reduced absorption around the GS edge absorption peak (feature b) followed by the increased absorption (feature c) indicates the blue shift of the absorption edge energy. The extra 1s to 4p transition and the blue shift of the edge energy in the XAS spectrum of PEABr thin film unambiguously demonstrated photo‐generated holes are getting localized at Br center and staying up to 100 ps delay. This agrees with the extended PL lifetime measured in Figure 2e,f for PEABr thin film. However, in the case of BABr thin film, the charge carriers are already recombined at early time through non‐radiative pathways based on the PLQY analysis.

Combining the optical spectroscopy and X‐ray absorption characterizations on the PEABr and BABr thin films, we conclude that in the PEABr thin film, charge carriers live longer because of the charge exchange between shallow traps and the band edge. This leads to the observed charge localization at the bromide 4p orbital or shallow traps, which in turn greatly enhances the chance of the radiative charge recombination yielding a high PLQY. In the beginning of our study, the GIWAXS maps (Figure 1b,c) revealed similar macroscopic crystalline structures in the PEABr and BABr thin films. The film morphology and crystallite sizes are investigated in greater details by scanning electron microscopy in Figure S3, Supporting Information, that also suggest the crystalline structures are comparable in both of the films using this processing condition. No significant stokes shifts in Figure 1d,e were observed in either case. Further, the local bond structure near Br site is also similar in the two films based on the X‐ray absorption ground state spectra. Therefore, the distinct emission efficiencies are attributed to the variation of intrinsic electronic properties of the semiconductor altered by the organic spacers in the thin film.

After describing the photo‐physical process and charge carrier recombination routes in the thin film, we further aim to systematically characterize our RPLPs LEDs (**Figure** **4**) to understand the device operational mechanism in connection to the physical properties. The luminance‐bias characteristics plotted in Figure 4a compare the PEABr and BABr devices. The average luminance for a typical PEABr device reaches 6.6 × 10^4^ cd m^−2^ in contrast to a BABr device reaching only ≈10^3^ cd m^−2^. Besides, the PEABr device shows a lower turn‐on voltage at around 3.83 V while that for the BABr device is 5.5 V (Figure S6, Supporting Information). Because of the high luminance and current efficiency, the external quantum efficiency of the PEABr device is around 9.2% ± 1.43% as averaged over 30 devices (Figure 4b). To probe the device characteristics more carefully, we plot the radiance (*R*) curves along with the current density (*J*) curves with applied bias (*V*) in Figure 4c,d and the radiance and EQE as a function of injection current density in Figure 4e,f. Comparing the *J*–*R*–*V* curves of the PEABr (Figure 4c) and BABr (Figure 4d) devices plotted in the same scales, it is clear showed that while the injected current density are comparable in both of the devices, the brightness rises drastically faster after being turned on in PEABr device as compared to that for the BABr device. Thus, the radiance of the PEABr device increases synchronously along with the injection current density suggesting an efficient radiative recombination of the injected carriers. In contrast, the BABr device's brightness increases slowly after turning the device on, and finally levels to much lower value. Consequently, the BABr device shows appreciable brightness and quantum efficiency only at much higher injection current, whereas the PEABr device demonstrates much higher value right after the turn‐on current (Figure 4e,f). In Figure 4f, we note the quantum efficiency of the PEABr device increases monotonically with the injection current density, and the peak quantum efficiency of the PEABr device close to 11% corresponds to the device with a high brightness of 7.51 × 10^4^ cd m^−2^.

**Figure 4:**
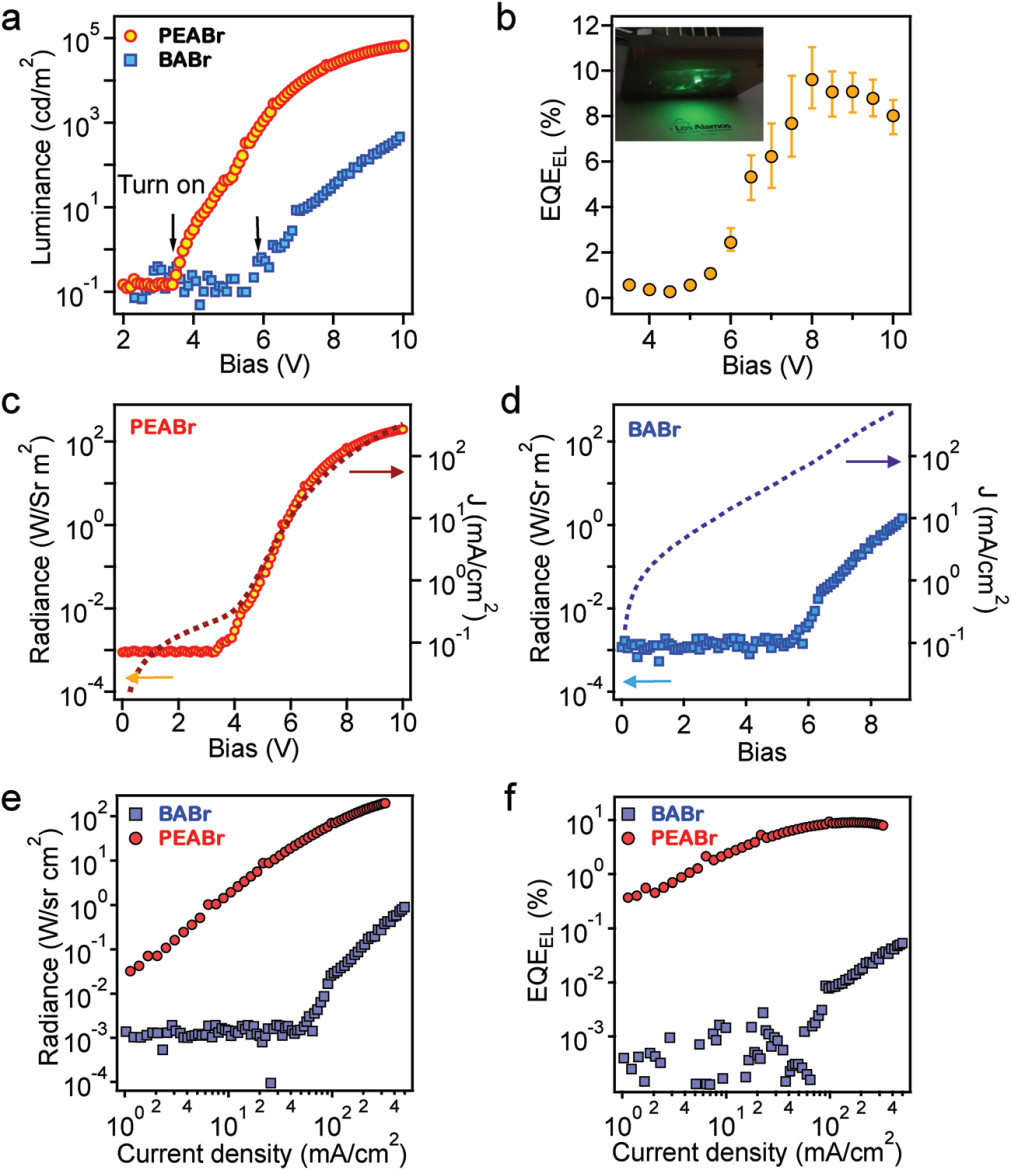
LEDs device characteristics. a) the luminance for the PEABr and BABr devices as a function of operational bias. b) the average external quantum efficiency for the PEABr devices as a function of applied bias. Inset is the photograph of a device operating at 6 V. The average is taken across 30 devices fabricated at the optimized conditions. c,d), The radiance–voltage and current density–voltage curves for the c) PEABr and d) BABr devices. e,f), The radiance–current density and EQE–current density curves, respectively, for the PEABr and BABr devices.

From the device characteristics, we single out several features inherent to the PEABr device and contrasting to the BABr device, which contribute to the much higher EL efficiencies for the former: a) The PEABr device turns on at much lower voltage (Figure 4a) and injected carrier recombination (Figure 4e) is efficient, leading to light emission in the low injection regime. b) As the injection current density increases, the brightness rises monotonically in the PEABr device (Figure 4c), while that increases slowly in BABr device (Figure 4d). This indicates that most of the carriers injected into the BABr device are lost through the non‐radiative decay path thus diminishing electroluminescence. These device characteristics can be readily rationalized by the photo‐physical properties of the materials shown in Figures 2 and 3. In PEABr, the charges live longer being localized in the shallow traps near the band edge, which dynamically exchange with the delocalized band‐edge state opening path to radiative recombination. On the other hand, the BABr device loses the injected carriers potentially through deep traps that lead to a much dimer emission when driven at comparable injection current. Finally, we encapsulated the PEABr device with UV‐curable epoxy and cover glass for operation lifetime test. The EL half‐lifetime (*T*_50_) under continuous operation with 8 V bias (or 98 mA cm^−2^) in the ambient condition reaches 25 min (Figure S7, Supporting Information).

In this study, we exhaustively compared two type of Ruddlesden–Popper phase layered perovskite thin films with alkyl chain (BA) and benzyl ring (PEA) as organic spacers for light emitting applications. In both cases, free carrier recombination is the dominating charge recombination mechanism. With the PEA spacer in the lattice, the charges are relatively long‐lived and localized near the band edge through thermal exchanging with shallow traps, without being captured by the deep traps. This is supported by the relative PLQY and the power dependent carrier dynamics characterizations. With the longer lived carriers, the radiative recombination is facilitated with higher PLQY. In the atomistic level, we directly observe that the charges are localized at the states composed of Br 4p orbitals in the PEABr thin film as evidenced by the X‐ray absorption spectroscopy measurements. The Br 4p orbitals form the valance band edge as well as part of the shallow trap states. Such observation indicates that the long‐lived charges in PEABr thin film are localized near the band edge (or shallow trap very close to the band edge) at longer time (>100 ps). However, the charges in the BABr films decay much faster through non‐radiative pathway and therefore no perturbation near the band edge was detected at 100 ps after charge generation. Our experimental results suggest that employing alkyl chain and benzyl rings at R′ site in the layered perovskites leads to a large difference in charge recombination pathways. We propose several structural mechanisms that can be the origins for such a drastic difference in the two films. One possibility can be the steric hindrance (volume) of the organic molecules in layered perovskites, similarly proposed in the 3D perovskite cases.^29^ The benzyl rings at R′ site occupy larger volume and thus may influence the crystalline packing of the inorganic cages. While the alkyl chain is more flexible that does not affect the band structures in the layered perovskite. It is also proposed the pi‐electron at the benzyl group may conjugate with the neighbor electron that affects the molecular packing in the system.^30^ Both structural change by the R′ organics could introduce shallow traps into the system. Other possible origin could be the molecular rotation in the lattice.

2D bromide perovskites are recognized as excellent emitter for light emitting diode applications. Great efforts have been devoted in the Cs‐based nano‐structured perovskite LEDs with stable operations,^8^ while the 2D hybrid perovskites also show advantages in the performances.^11^, ^12^, ^15^, ^31^ Our study suggests that the choice of organics is critical to obtain highly emissive 2D perovskites, where PEA cation included 2D hybrid perovskites show extended lifetime and large degree of localization that promote the radiative recombination. Interestingly, in Cs‐based 2D perovskites, the BA incorporated materials are more emissive.^16^, ^32^ While the thin film crystalline quality for the PEABr and BABr thin films in our study are comparable evidenced by GIWAXS and SEM image analysis, the PEABr film contains more low n‐valued perovskite phase judging from the absorption and PL spectrum. Along with the observed longer PL lifetime in PEABr films, we conclude that the deep defect is greatly suppressed in the PEABr sample. It was observed in previous reports^33^ that higher PEABr/MABr ratio in the quasi‐2D perovskite thin films resulted in higher emission yield with extended PL lifetime.

In addition to the phase purity discussion, it has been shown in several reports that bulky organics tend to rotate in the lattice that causes strong electron‐phonon interaction in 2D perovskite system that improves the emission efficiency.^18^, ^19^ In our case, apart from the fact that the PEABr film has a cleaner 2D phase perovskite, the electron‐phonon coupling cannot be excluded as a possible reason for the observed charge localization, when the rigid benzyl rings rotate in the lattice and influence the octahedron cages more dramatically that lead to shallow trap formation. Other possibilities such as intrinsic vacancy with very small energy below the gap that are present in benzyl ring containing perovskites cannot be excluded as well. All these proposed mechanisms point to the conclusion that the choice of the organic spacers in the layered perovskites can control charge recombination pathways.

## Experimental Section

##### Materials and Instruments

*N*,*N*′‐Bis(3‐methylphenyl)‐*N*,*N*′‐diphenylbenzidine (TPD), butylamine (BA), phenylethylamine (PEA), methylamine hydrochloride (MACl), lead (II) oxide, hydrobromic acid (HBr), hypophosphorous acid (H_3_PO_2_), 2,2′,2″‐(1,3,5‐benzinetriyl)‐tris(1‐phenyl‐1‐*H*‐benzimidazole)(TPBi), and dimethylformamide (DMF) were purchased from Sigma‐Aldrich and used as received. Calibrated silicon diode (FDS100‐Cal, Thor Labs), Ocean Optics (USB 4000), Kiethley 236, and Keithley 2400 were used for current voltage characteristics and radiance measurement.

##### Thin Film Sample Preparation

The 2D RPLPs perovskite thin films samples for optical measurements and characterizations were prepared on pre‐cleaned glass substrate with 0.125, 0.25, and 0.5 m of Pb^2+^ concentration precursor solution then spun cast with spin speed at 5K rpm for 20 s, followed by post annealing at 110 °C on hot plate for 10 min.

##### Devices Fabrication and Characterization

The 2D RPLPs precursor solutions were prepared with 0.25 m of Pb^2+^ concentration and stirred at room temperature for overnight followed by previous report. RPLPs LEDs devices were prepared with ITO/TPD/2D RPLPs/TPBi/Al architecture. The RPLPs emitting layers were fabricated using the same method as described in thin‐film characterization. The TPD (40 nm), TPBi (40 nm), and aluminum top electrode (100 nm) were prepared by thermal evaporation in vacuum chamber. The area of 0.04 cm^2^ was used to define the device working area. All devices were prepared and used UV‐curable epoxy to protect the electrodes with a small‐area cover glass. The perovskite thin‐films for optical measurements were prepared on glass substrate using the same method as described earlier. The EL spectra of 2D RPLPs LEDs were collected by Ocean Optics. The radiance/EQE data were collected with a previously report method.^34^ Generally, by applying voltage to devices and collecting radiance value by the calibrated large area silicon diode and further calculated the EQE values. The calibrated large area silicon diode was kept at a fixed distance from the testing device and the solid angle could thus be calculated from the area of the cell, distance, and the area of the diode.

## Conflict of Interest

The authors declare no conflict of interest.

## Supporting information

Supporting Information
