# Supplementary material for: Critical Role of Organic Spacers for Bright 2D Layered Perovskites Light‐Emitting Diodes
Source: Adv Sci (Weinh). 2020 Feb 19;7(7):1903202. doi: 10.1002/advs.201903202 (PMC7141028; doi:10.1002/advs.201903202)
Supplement: Supplementary file 1 — Supporting Information [file ADVS-7-1903202-s001.pdf]

Copyright WILEY-VCH Verlag GmbH & Co. KGaA, 69469 Weinheim, Germany, 2020.

ADVANCED SCIENCE
Open Access

Supporting Information

for *Adv. Sci.,* DOI: 10.1002/advs.201903202

Critical Role of Organic Spacers for Bright 2D Layered Perovskites Light-Emitting Diodes

Hsinhan Tsai, Cunming Liu, Eli Kinigstein, Mingxing Li, Sergei Tretiak, Mircea Cotlet, Xuedan Ma, Xiaoyi Zhang, and Wanyi Nie**

Copyright WILEY-VCH Verlag GmbH & Co. KGaA, 69469 Weinheim, Germany, 2020.

Supporting Information

Critical Role of Organic Spacers for Bright 2D Layered Perovskites Light-emitting Diodes

Hsinhan Tsai*, Cunming Liu, Eli Kinigstein, Mingxing Li, Sergei Tretiak, Mircea Cotlet, Xuedan Ma, Xiaoyi Zhang, Wanyi Nie*

1. Materials synthesis and characterization

Generally, it combined PbO, MACl, RAI (R=BA and PEA) with proper molar ratio in HBr/H_3PO_2 mix solution and heat to 170 ºC with constant magnetic stirring bar for slowly evaporate the solvent. When the perovskite flakes start precipitate, quickly remove the solution from hot plate and cool at room temperature for overnight. PXRD was used to characterize the purity of as synthesized crystals and determined the number of layers in 2D RPLPs as presented in Figure S1. From PXRD data, we are mainly focus on ($0k0$) peaks in low angle regim. For n=3 layered perovskites as example, we should expect three equally spacing ($0k0$) peaks in low angle regim. Clearly, We successfuly synthesize with pure-phase n=3 layered perovskites with PEA and BA as large organic spacers.

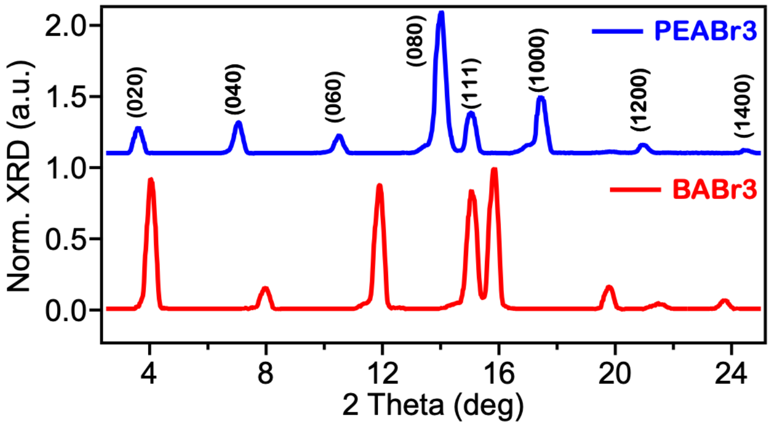

Figure S1. Powder X-ray diffraction pattern for phase purity characterization of n=3 layered perovskites with PEABr(red) and BABr (blue) as organic spacers. The insets are the photograph of as-synthesized crystals.

2. Grazing incidence wide-angle X-ray scattering (GIWAXS) characterization

Synchrotron grazing incidence wide-angle X-ray scattering (GIWAXS) measurements was performed at Beamline sector 8-ID-E of the Advanced Photon Source (APS) at Argonne National Laboratory. GIWAXS samples were placed in the rotary sample stage under 10^{-3} torr vacuum chamber and exposed to an X-ray beam (λ = 1.6868 Å) at an incident angle of 0.20° for 5 s, and the scattered light was collected by a Pilatus 1 M pixel array detector at 204 mm from the sample. The GIWAXS data were processed with GIXSGUI package for Matlab (Mathworks) with correction for detector sensitivity, X-ray polarization, and geometrical solid angle.

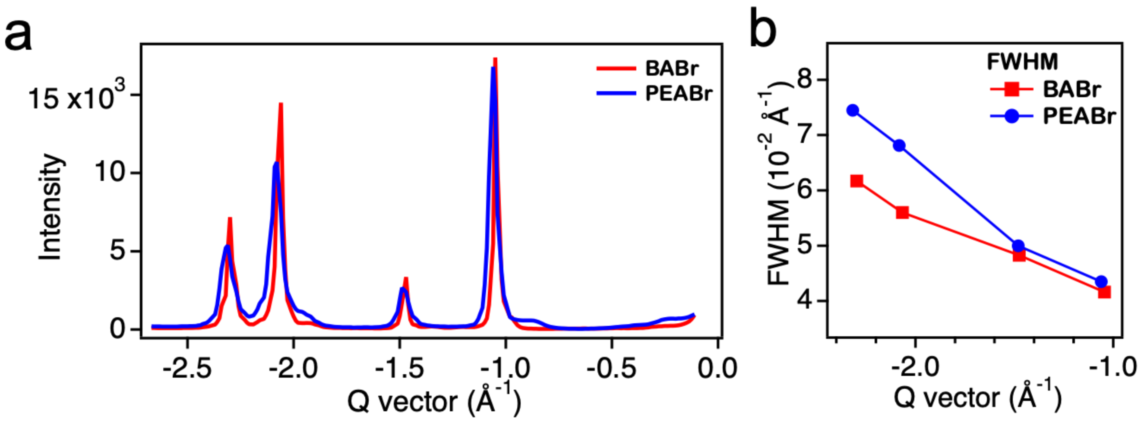

Figure S2. GIWAXS data analysis. (a) Line-cut and (b)FWHM for PEABr and BABr thin film samples.

3. Surface morphologies for PEABr and BABr thin film

A scanning electron microscope (FEI inspect quanta 400) images were obtained with spot size 3 and 5 KeV for surface morphology characterization.

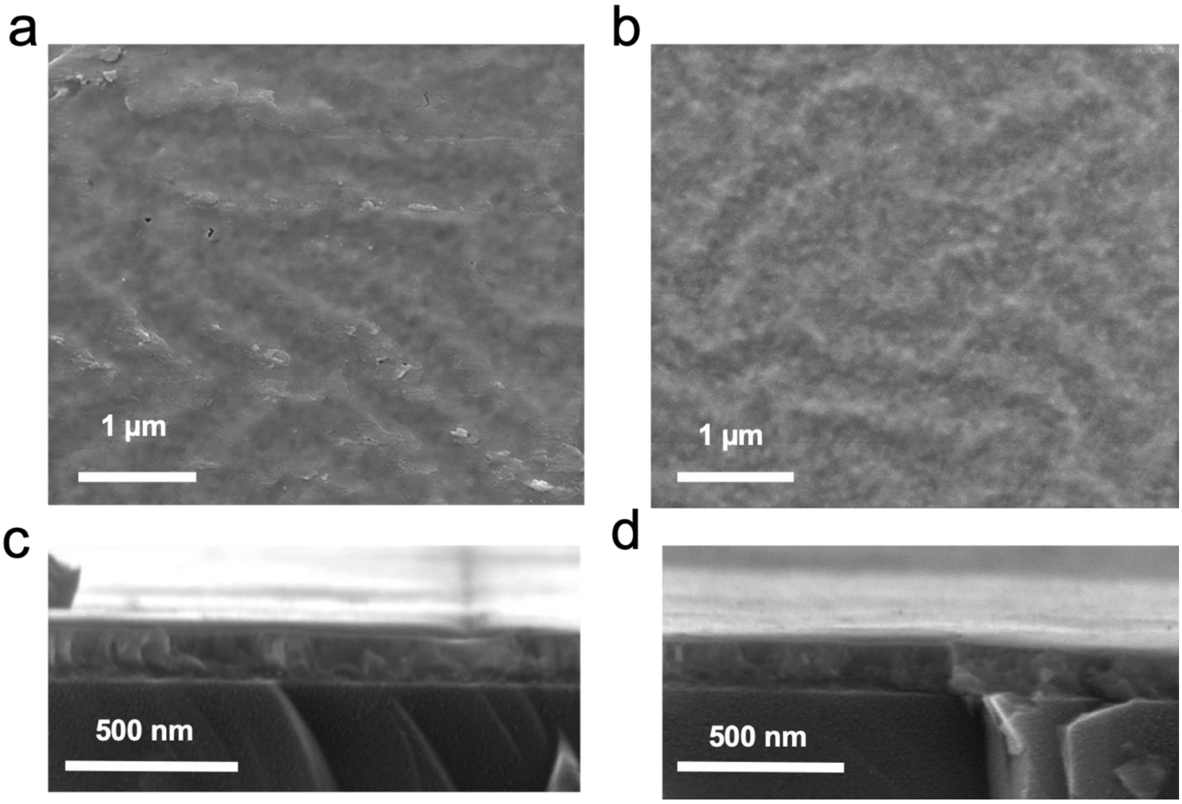

Figure S3. SEM images for surface and cross-section mophologies of (a) BABr(a, c) and (b) PEABr(b,d) thin films.

4. Absorption/PL mapping/PLQY measurements.

Absorption spectra of the thin films were measured using a UV/Vis spectrometer. PL mapping measurements of the films were performed on a home-built confocal laser microscope. Pulses from a 400 nm diode laser with a frequency of 1 MHz were focused by a microscope objective onto the samples. PL from the films was collected by the same objective, cleaned up by long pass filters, and sent to a single-photon avalanche diode for constructing scanning PL images and PL timetraces. To calculate the PLQYs, PL intensities of the two types of thin films measured under exact same conditions were normalized to their absorbance at the excitation wavelength.

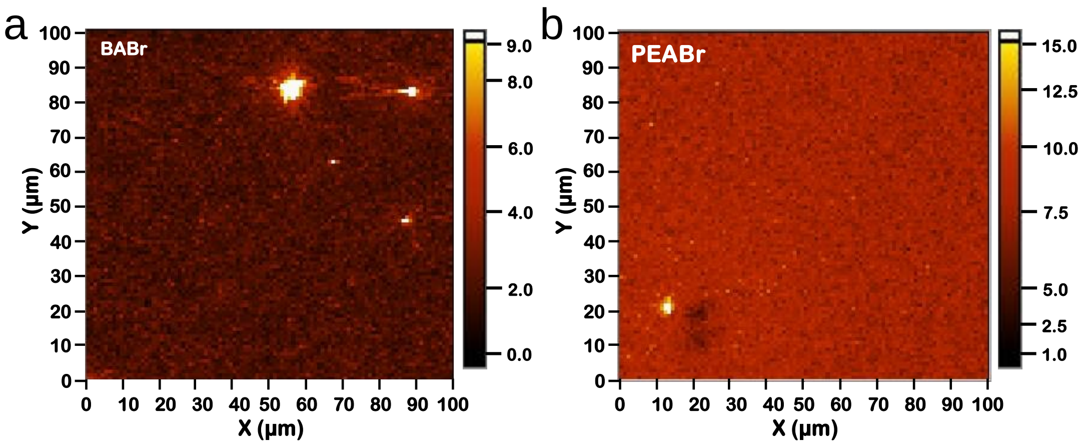

Figure S4. Large area PL mapping for **a,** BABr and **b,** PEABr thin films under same laser power.

5. Time-resolved photoluminescence (TRPL).

Time-resolved photoluminescence measurements were performed on a home-built inverted microscope (Olympus IX81, 0.90 NA 60× air objective) coupled to a 375 nm pulsed laser system (85 ps pulse width, Spectra Physics) operated at a 4 MHz repetition rate. Photoluminescence was collected in epi-illumination scheme, spectrally separated from the excitation laser light by a dichroic (Semrock, FF390-Di01) and by a band-pass filter according to the position of photoluminescence peak, spatially filtered by a 100 μm pinhole and then imaged onto a single photon counting avalanche photodiode (MPD Picoquant) coupled to a time-analyzer (PicoHarp 300, PicoQuant). Data acquisition and data analysis were performed with the Symphotime 64 analysis software (Picoquant). The PL is fitted by three exponential equation. The intensity average lifetime is defined as . The PL decays in this study were recorded as PL intensity vs time. Therefore, the fitting directly reflected the intensity of each components.

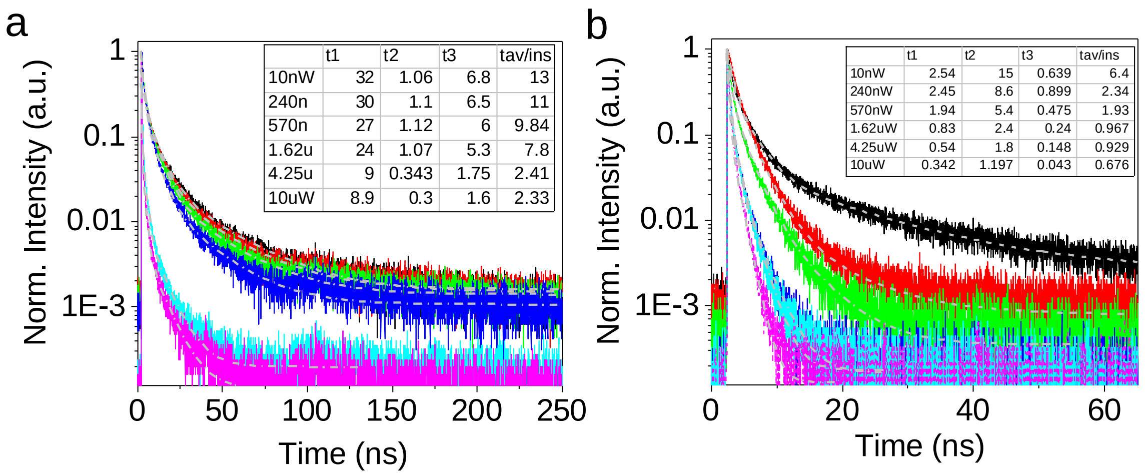

Figure S5. PL dynamic studies for **a,** PEABr and **b,** BABr thin films under various incident laser power.

6. Time-resolved X-ray absorption (TR-XAS) spectroscopy.

The TR-XAS measurements were performed at the beamline 11-ID-D of Advanced Photon Source (APS) in Argonne National Laboratory. The 400 nm, ~ 1.6 ps (fshw) laser pump pulse was the second-harmonic output from 10 kHz Coherent Legend Elite Ti: Sapphire ultrafast amplifier laser system. The X-ray probe pulses were derived from electron bunches extracted from the storage ring. The experiment was carried out under a hybrid timing mode where an intense X-ray pulse with 16% of the total average photon flux was separated in time from other weak X-ray pulses. The X-ray pulse was delayed by certain time to probe the sample after laser excitation. For constructing the X-ray absorption spectra, the X-ray fluorescence signals from Br atoms were collected at 90° angle of the incident X-ray beam by an avalanche photodiode (APD). A soller slits/Se for Br filter combination, which was custom-designed for the sample chamber configuration and the distance between the sample and the detector, was inserted between the thin film sample and the APD detector. The thin film samples were fast rotated (~ 1000 rpm) by a spinner in order to reduce/avoid photodegradation. The laser off trace ($\mu(E)_{laser\_off}$) representing the spectrum of the ground state (GS) species ($\mu(E)_{GS}$) was constructed by averaging the x-ray fluorescence signals from six X-ray probe pulses before laser excitation. The laser_on spectrum ($\mu(E)_{laser\_on}$ (Δt)) was

collected using the fluorescence signal from the X-ray pulse at the certain time delay Δt (e.g. 100 ps) after 400 nm photoexcitation of the sample, which contains the contributions of the excited state (ES) and ground state (GS) spectra ($\mu(E)_{laser\_on}$ (Δt) =(1- $\eta(\Delta t)$) $\mu(E)_{GS}$ + $\eta(\Delta t)$ $\mu(E)_{ES}$, where $\eta(\Delta t)$ is the fraction of excited state spectra at the time delay Δt) . Hence, the TR-XAS spectrum ($\Delta\mu(E)(\Delta t)$) can be obtained by subtracting $\mu(E)_{laser\_off}$ from $\mu(E)_{laser\_on}$ (Δt), namely $\Delta\mu(E)(\Delta t)= \mu(E)_{laser\_on}(\Delta t) - \mu(E)_{laser\_off}$.

7. Average turn-on voltage for RP PeLeds

The avaerage turn-on voltages statistic were collected from PEABr and BABr devices with randomly selected 30 devices.

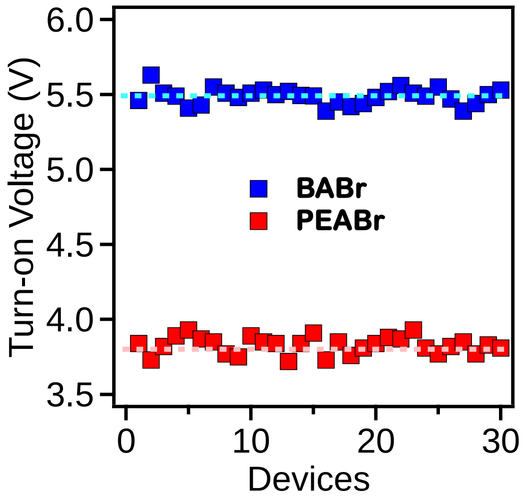

Figure S6. Average RPLP LEDs device turn-on voltage for BABr and PEABr over 30 devices.

8. Device stability test

The device operation lifetime for PEABr PeLEDs was using simple ultraviolet-curable epoxy resin with cover slide for encapsulation and was tested in air under 8V applied bias.

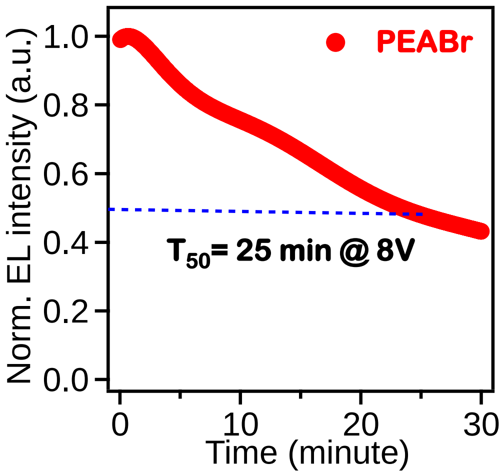

Figure S7. Devices stability test for PEABr device under constant applied bias at 8V.
